# Development and implementation of a longitudinal students as teachers program: participant satisfaction and implications for medical student teaching and learning

**DOI:** 10.1186/s12909-017-0857-8

**Published:** 2017-01-31

**Authors:** Celine Yeung, Farah Friesen, Sarah Farr, Marcus Law, Lori Albert

**Affiliations:** 1 Faculty of Medicine, University of Toronto, Toronto, ON Canada; 2 Centre for Faculty Development, University of Toronto, Toronto, ON Canada; 3 Rheumatology Clinic, Toronto Western Hospital, 399 Bathurst St, East Wing Room 1F-835, Toronto, ON M5T 2S8 Canada

**Keywords:** Students as teachers, Medical students, Learning by teaching, Program satisfaction

## Abstract

**Background:**

Teaching is a key component of medical practice, but medical students receive little formal training to develop their teaching skills. A longitudinal Students as Teachers (SAT) program was created at the University of Toronto to provide medical students with opportunities to acquire an understanding of educational pedagogy and practice teaching early in their medical training. This program was 7-months in duration and consisted of monthly educational modules, practical teaching sessions, feedback, and reflective exercises.

**Methods:**

A mixed methods study design was used to evaluate initial outcomes of the SAT program by obtaining the perspectives of 18 second-year medical students. Participants filled out questionnaires at the beginning and end of the 7-month program to indicate their skill level and confidence in teaching. Differences between pre- and post-intervention scores were further explored in a group interview of 5 participants.

**Results:**

Participants expressed a high degree of satisfaction with the SAT program structure and found the educational modules and practical teaching sessions to be particularly beneficial to their learning. Over the course of the program, there were significant increases in students’ confidence in teaching, and self-perceived teaching capacity and communication skills. Furthermore, participants discussed improvements in their effectiveness as learners.

**Conclusions:**

Teaching is a skill that requires ongoing practice. Our results suggest that a longitudinal program consisting of theoretical modules, practical teaching sessions, feedback, and reflective exercises for medical students may improve teaching and communication skills, and equip them with improved learning strategies. This program also provides students with insight into the experience of teaching while holding other academic and clinical responsibilities.

**Electronic supplementary material:**

The online version of this article (doi:10.1186/s12909-017-0857-8) contains supplementary material, which is available to authorized users.

## Background

Effective teaching is a skill that is developed through ongoing observation, training, and practice. While content expertise is often thought to translate into competency in teaching, recent findings suggest that the two do not directly correlate [1]. Given that teaching is a key component of medical practice from teaching students in the classroom to instructing patients in the clinical setting, it seems logical that physicians should be taught how to teach [2]. However, physicians do not routinely receive formal instruction or coaching to develop their teaching skills during their medical training. Dandavino and colleagues [2] have emphasized that teaching is a core clinical skill that should be taught in a sequential manner through ongoing training, practice, and feedback. As such, Students as Teachers (SAT) programs have been gaining attention as a way to address this important skill during undergraduate medical education.

SAT programs may have multiple benefits. Medical students who learn how to teach may improve their communication skills and their ability to educate patients [3]. Improvements in a physician’s ability to teach and communicate with patients may be directly associated with improved health outcomes for their patients [4]. Students who learn to teach may gain a greater appreciation for learning principles, thereby becoming better learners as they apply these theories to their own education [5]. Finally, the provision of early opportunities for professional development may encourage students to pursue similar opportunities during postgraduate years (e.g. Residents as Teachers programs or clinician-educator programs) [6].

Despite the potential benefits of a SAT program, there is surprisingly little research and literature examining longitudinal SAT programs for medical students. Most existing initiatives have had variable instructional formats, ranging from a few half-day workshops or 1–2 week electives with limited opportunities for practice [6, 7], to an intensive 7-week block in which students were immersed in teaching and were exempt from having clinical responsibilities or other educational demands [8]. While ideal, the latter scenario may not be feasible in all medical school curricula, and may not reflect the real-world demands of a physician at an academic centre. Furthermore, almost all programs are designed for third or fourth year medical students to prepare them for teaching during residency or to help alleviate the teaching duties of academic physicians at institutions where clinician-teachers are scarce.

The purpose of the current study is to describe the development, implementation and evaluation of an extracurricular longitudinal 7-month SAT program for 20 second-year medical students at the University of Toronto. Unlike other initiatives, this program was integrated into the full academic year, requiring students to juggle other educational tasks, which may better mimic the demands of an academic physician. Furthermore, the program was introduced at the pre-clerkship level as an opportunity for students to begin developing their teaching skills early on. Here we report our initial outcomes with respect to participants’ satisfaction with the program, perceived teaching capacities, and personal learning behaviours.

## Methods

### SAT program participants

Among 41 applicants, 20 medical students were selected to enter the SAT program through a blinded application process (11 females and 9 males; ages: 22-28). The only eligibility criterion was enrolment at the University of Toronto as a second year medical student, and students were selected based upon their interest in medical education or desire to improve their teaching skills. This was assessed through an online application. Applications were reviewed by three faculty members who were not involved in the research study. Many of the students who applied had prior teaching or tutoring experience, which ranged from being a teacher in the community (e.g. swimming instructor) to being a formal undergraduate teaching assistant. However, teaching experience was not required for entry into the program.

### SAT program curriculum

The curriculum was designed by a group of faculty and medical students based on evidence from the medical education literature, and a needs assessment survey that was sent to a diverse group of medical students. The program consisted of eight 2-h educational modules, five practical teaching sessions, and three independent assignments [see Additional files 1 and 2]. The program lasted 7 months with one or two modules per month, and one practical teaching session in between each module for students to apply the concepts learned. The three independent assignments were submitted as summative pieces at the end of the first and second academic terms. The program was designed to be an extracurricular activity and did not contribute toward the students’ academic grades. Participants worked towards achieving a certificate of completion, which required mandatory attendance of the first and last modules, at least 5 of 6 remaining modules, and completion of all teaching sessions and independent assignments. The overall mean percentage of attendance/completion of program components was 82%.

#### Educational modules

The content for the modules was selected based on a needs assessment survey that was sent to all medical students interested in the program to determine what teaching principles they were eager to learn. Evidence from existing SAT programs at other institutions was also considered [6]. Module topics included ‘Orienting the Learner’; ‘Principles of Adult Learning’; and ‘Small Group Teaching’. The modules were interactive sessions, comprised lecture and group activities, and were led by experienced clinician-educators who had facilitated similar sessions for health care professionals in pre-existing professional development programs. These sessions included discussion of medical education theories as well as practical teaching strategies (e.g., how to set learning objectives).

#### Practical teaching sessions

For three of the five sessions, students were split into small groups and engaged in peer-to-peer teaching on a topic related to any aspect of medicine. This structure mirrors a predominant method of teaching on the wards [8]. For the remaining two sessions, students taught individuals outside of the SAT program in a small and large group setting. For the latter, students worked in their small groups alongside a medical education expert (clinician or researcher) to prepare a presentation. They then delivered this talk to a large audience (>40 students and two clinician educators). All performances were videotaped and each participant completed a self-assessment form and provided feedback to their peers.

#### Independent work

Participants completed two reflective exercises, commenting on teaching strategies they observed in different learning environments (small vs. large group; clinic vs. operating room). Students also consolidated their experience in the program by creating a teaching dossier.

### Study design

A mixed methods study design was used to evaluate 18 program participants (second-year medical students) between October 2014 and July 2015 at the University of Toronto. Participants filled out questionnaires at the beginning and end of the program to indicate their skill level and confidence in teaching. Differences between pre- and post-intervention scores were further explored qualitatively in a group interview of five randomly selected participants.

Recruitment letters for the study were sent to all program participants, except for one student who was involved with the study. Eighteen students provided informed consent and agreed to participate, while one student declined to participate. A third party with no affiliation to any of the participants gave each individual a code to ensure that responses remained anonymous to the investigators. The participants were asked to use the code while filling pre- and post-questionnaires such that individual change in self-assessment could be measured. Potential conflicts of interest were determined a priori, disclosed to the research ethics board, and addressed with strategies during the data collection process. For example, given that some participants knew the primary investigators, all identifying information was removed from the questionnaires and the group interview was conducted by a neutral third party (research associate). Approval for the study was received from the University of Toronto Health Sciences Research Ethics Board (Protocol #30819). All participants also consented to having their data published.

At the beginning of the program, all study participants completed an online pre-questionnaire. This questionnaire consisted of text-based and Likert-scale type questions to determine participants’ demographic information, baseline skill level, confidence in teaching, self-perceived teaching capacity and communication skills. At the end of the program, participants completed an online post-questionnaire to determine their level of satisfaction with the program, and any change in their confidence in teaching, self-perceived teaching capacity and communication skills.

Following the post-questionnaire, five students were selected using a purposive sampling method to participate in a group interview [9]. One student from each SAT small group was selected to generate a diverse collection of various perspectives, as each small group had different dynamics that may have affected participants’ overall experience in the program. Furthermore, participants in the interview were asked to reflect on their interactions with their group members, which could have led to censorship if more than one group member had been present for the interview. The purpose of the interview was to elicit more descriptive responses that may not have been captured by the questionnaire. The interview was conducted in a private classroom, lasted 1 h in duration, and was audio-recorded with the consent of participants. Audio recordings were manually transcribed within 5 days of the interview.

### Statistical analysis

To determine whether a difference existed between pre- and post-intervention scores, a two-way repeated measures ANOVA and a Wilcoxon signed rank test were performed for continuous and ordinal paired data, respectively. All statistical analyses were performed using GraphPad Prism version 5.03. The group interview was coded by three investigators (researcher triangulation [9]) using NVivo 10 (QRS International, Burlington, MA). The data was then analyzed using an inductive approach, where common, emerging themes were identified from the three sets of codes [9].

## Results

### Questionnaire results

#### Students expressed a high degree of satisfaction with the SAT program structure

Baseline demographics of participants are shown in Table 1. In terms of program satisfaction, 94.5% and 88.9% of respondents agreed or strongly agreed that the educational modules and practical teaching sessions were helpful to their learning, respectively. This was followed by the debrief sessions (72.2% agreed or strongly agreed) and the teaching dossier assignment (61.2%), while reflective exercises (50%) were considered the least useful component (Fig. 1a). Overall, >75% of respondents agreed or strongly agreed that there was a sufficient number of each program component to help them gain theoretical knowledge in medical education and practical teaching experience (94.4% agreed or strongly agreed that there were sufficient modules, 88.9% for practical teaching sessions, 83.4% for reflective exercises, and 77.8% for debrief sessions) (Fig. 1b).

**Table 1:** Demographics of study participants

	# of study participants
*Gender*	
Male	9
Female	9
*Age*	
20–22	2
23–24	12
> 25	4
*Education*	
Bachelors	14
Masters	3
PhD	1

**Fig. 1:**
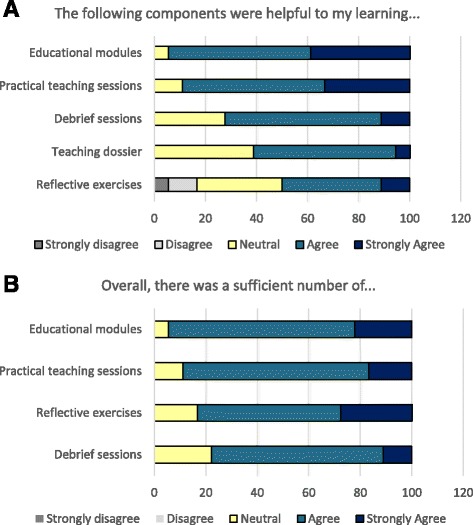
Students expressed a high degree of satisfaction with the SAT program structure. The following outcomes were assessed to determine participant satisfaction with the SAT program structure: **a** Students’ perception of how helpful different components of the SAT program were to their learning. **b** Students’ perception of whether there were a sufficient number of the various components of the SAT program

#### Participants’ confidence in teaching, self-perceived teaching and communication skills, and ability to engage in feedback and reflection increased significantly

**Fig. 2:**
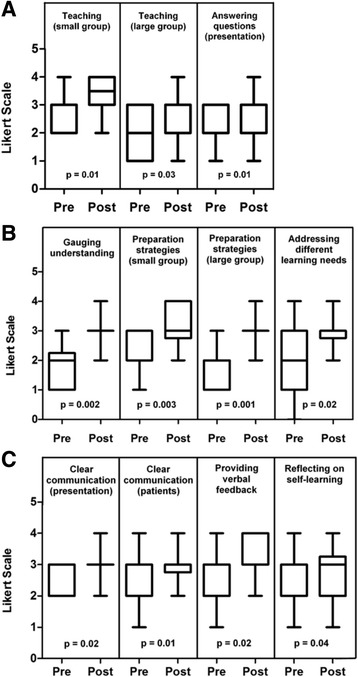
Participants’ self-perceived confidence, teaching skills, and communication skills increased throughout the program. The following outcomes were assessed among participants prior to (pre) and following completion of the SAT program (post): **a** participants’ confidence in teaching in small and large group environments and in answering questions during or after a presentation; **b** participants’ perception of their teaching skills in the following domains: their ability to gauge learner understanding, prepare for small and large group teaching sessions, and address learner needs; **c** participants’ perception of their communication skills in the context of delivering a presentation and when talking to patients, as well as participants’ perception of their ability to provide verbal feedback and to reflect on their own learning

Participants’ confidence in small and large group teaching and in answering audience questions were significantly increased by the end of the program (Fig. 2a). This is in line with significant improvements in their self-perceived teaching skills, which included skills like their ability to gauge audience understanding, prepare for small and large group teaching sessions, and address different learning needs (Fig. 2b). Respondents also reported having stronger communication skills while delivering a presentation or speaking with patients, feeling more comfortable providing verbal feedback at the end of the program, and became more likely to reflect on their effectiveness as learners (Fig. 2c).

### Group interview results

Overall, participants expressed that the SAT program was a positive experience and saw the value of receiving early exposure to teaching in their medical training. There was a general consensus that teaching is an integral part of medicine given the role that physicians have in training junior colleagues and communicating with patients. Indeed, one student commented on the lack of formal programs preparing medical students for these future teacher roles:

“Kind of interesting when you think of the way our education is layered… because it’s like residents teaching clerks, fellows teaching residents, that maybe teaching is a pivotal role in our education process.”

“I’ve always found it interesting that we don’t receive any formal education training. Because teaching is so much a part of what we’re going to be doing in the future…”

Participants saw the SAT program as an opportunity to foster these teaching skills and to support their interests in teaching. They enjoyed learning from clinician-educators and found the practical sessions useful for applying the theories learned. Participants described the program as a “launch pad” to pursue other professional development programs and as a resource that they could use later on. They also noticed improvements in their teaching abilities and were able to identify strategies that were helpful in their own learning as students. However, participants felt that the program did not help with patient communication despite there being a significant increase in the participants’ perceived ability to communicate with patients in the questionnaire.

Three major themes emerged from the results of the group interview.

#### Theme 1: A longitudinal SAT program increased perceived knowledge of educational theory and provided students with opportunities to practice teaching

The first theme that emerged was the utility of the SAT program in increasing perceived knowledge of educational theory. Participants saw the SAT program as a “toolkit” that provided them with approaches and strategies on how to teach in various settings.

“The SAT program was useful for me to help me think about how I should approach teaching someone and the different aspects of being a learner as well. For example, orienting the learner and making sure [of] their understanding of my role, their role, and how we interact with each other. I think all of those things are very valuable to me to become a teacher in the future.”

They referenced several theories that had been taught in the educational modules, demonstrating their enhanced knowledge base. They also discussed how they applied these theories during their teaching sessions. This aligned with an overall sense of improvement in participants’ teaching skills, attributed in part to the opportunities that the program provided to practice teaching.

“I think my favorite part of the SAT program was the practice – the hands on experiences that we got…what we were taught in the modules, we got to apply it in the activities.”

“I’m now cognizant of setting learning objectives and making sure that I’m always engaging my audience by asking them questions and making sure that I’m actually talking on their same level. I think having those practice sessions helped in terms of practicing these kinds of skills.”

#### Theme 2: A longitudinal SAT program allowed students to provide and receive feedback, and gave students opportunities to reflect on their practice

Participants valued the feedback and reflection components of the program as contributing to their enhanced teaching capacity. They appreciated opportunities to receive feedback from their peers, but also saw the benefit of learning how to provide effective feedback to others.

“Now I have an actual framework that I can run through whether it’s giving feedback to a lecturer or people that I’m working with…it’s definitely been very helpful.”

Similarly, reflective exercises encouraged them to think about their experiences and led to a greater appreciation for effective teachers in their own education. In fact, some participants felt that incorporating more reflections, such as self-evaluating their videotaped teaching performances or examining their videos alongside an expert, would strengthen the program even further.

“You have to carve out time [for reflection] because it’s a requirement [of the SAT program]…so in that sense I think it did help us to reflect on our own identity as teachers and learners and so I think it was helpful in that sense for sure.”

By having formal opportunities to practice teaching and engage in feedback and reflection, participants ultimately expressed an increase in confidence with teaching within themselves and among their peers.

“[SAT was] a confidence booster…[it] made me feel like I got some practice and learned some things and now I think I might be a little bit more effective than I was when I first started.”

“I could definitely see the confidence that boosted people who were in my group…they incorporated some things that were taught in the sessions, like learning objectives and conflict of interest slides.”

#### Theme 3: A longitudinal SAT program equipped students with improved learning strategies

Participants maintained a strong identity as students and described how the program improved their own learning behaviors. Their narratives focused on the ways in which concepts taught in the SAT modules could be used to direct their own learning. For example, participants developed a greater appreciation for the role of clear, meaningful learning objectives:

“There were certain times during the year, like right after we had the objective module, I went into a lecture and was like “why don’t you cite the objectives?”…I think that definitely made me more aware of areas of improvement that some of our lecturers could have made. As for our own learning, I think it also helped me identify some ways to specify exactly what I wanted to learn – set better objectives for myself.”

Another example relates to students learning about the Johari Window during their Effective Feedback module, a technique that can improve the student-preceptor relationship. One student was encouraged by this learning to improve her communication with her preceptors, making her knowledge gaps more explicit, and helping the preceptors to better understand her baseline knowledge:

“One concept in particular which really impacted me was the…Johari window concept and the idea that the person who’s teaching doesn’t know what you know…I think that’s something I’ve done while shadowing is trying to be really clear about [what] we haven’t covered…instead of nodding along…[it] has allowed me to accept that it’s okay not to know things, and by communicating that it makes your learning a lot better in that environment.”

## Discussion

A longitudinal 7-month SAT program was designed and implemented at the University of Toronto as an extracurricular initiative for 20 second-year medical students. The present study evaluated initial outcomes of this SAT initiative following the first year of its implementation, including perceived teaching capacity, learning behaviors, and satisfaction with program design.

Consistent with previous studies assessing peer-to-peer or near-peer small group teaching, SAT program participants experienced a significant improvement in their confidence in teaching according to both questionnaire results and the group interview. This increase in confidence may have developed through the many practice and feedback sessions that students regularly engaged in with their small groups, as learning has been suggested to occur through experiential opportunities [10] and ongoing feedback [11]. Furthermore, the longitudinal format of the SAT curriculum allowed students to receive constructive criticism, as well as time for them to improve their approach through the practical teaching sessions.

Participants also felt that they acquired more strategies to prepare for their teaching sessions. This is supported by a previous study in which peer tutors reported having more preparation techniques after a 3-week training program [12]. The practical teaching sessions in the SAT program were designed to be a graded experience, from teaching in a small familiar group of peers to giving a large group lecture to an unfamiliar crowd that included experts. This increasing sequence of high-stakes experiences may have allowed students to gradually become more confident in their preparation abilities, and is consistent with Vygotsky’s constructivist theory that learning occurs when students leave their comfort zone and improve their skills by working with more capable, experienced individuals [13]. Indeed, for the large group teaching session, students worked directly with a clinician-educator or researcher to prepare their presentation. This graded experience may explain why participants reported having more small and large group preparation strategies by the end of the program.

In line with similar programs [14] and the various practical opportunities that SAT students received, participants reported significant improvements in their ability to communicate clearly while delivering a presentation. Interestingly, however, there was also a significant change in participants’ perceived ability to communicate with patients according to questionnaire results. This was further explored during the group interview. Despite the acknowledgement that teaching is an integral part of medicine, there was a general consensus among interviewees that the SAT program did not help them to improve their communication skills with patients. They felt that they did not consciously apply the teaching skills learned through the SAT program in this clinical context. This may have resulted from minimal exposure to patients and limited opportunities to apply their teaching skills in the clinic at this stage of their medical education. The significant difference observed in questionnaire results may have resulted from participants’ simultaneous involvement in a clinical skills course that is often a mandatory and common component of any undergraduate medical education program. A comparison with non-SAT students may be an objective method to determine whether the program actually contributed to this improvement. This observation requires further exploration and may support the notion that teaching skills developed through the SAT program are transferable to the clinic, independent of whether students are able to actively recognize this at their current stage of training.

In addition to improving their confidence and teaching skill level, participants in the group interview also described how teaching strategies taught in the SAT program improved their own learning behaviours (e.g. setting personal learning objectives or communicating gaps in their knowledge with their clinical preceptors). These findings support previous literature describing the ‘tutor learning effect’ [15] or the ‘learning by teaching’ model [16, 17], which emphasizes that learning is improved through the act of teaching. Students are not only able to develop their practical skills but also gain theoretical knowledge through the process of preparing for teaching sessions, verbalizing information, and answering questions [17]. Teaching forces information to be manipulated in different ways, which allows the student/teacher to monitor their own learning by identifying gaps in their knowledge and correcting previous misconceptions [17]. In fact, medical students who taught their peers were found to outperform those who were taught by experienced physicians [18], and students who studied material with the intention of teaching outperformed those who studied for the purpose of test-taking [16]. Our findings support this literature by demonstrating that students are able to apply teaching strategies to their own learning. Future studies should explore whether students who have learned how to teach are able to academically outperform those who have not developed their teaching skills.

Finally, there was a high degree of satisfaction among participants regarding the SAT program’s structure. The educational modules and practical teaching sessions were highlighted as particularly favourable components. Participants also appreciated the longitudinal format of the program and saw the value of learning these skills before their senior year of medical school or residency when they may be required to teach in the wards. Though the reflective exercises were not ranked highly, participants still acknowledged their utility and even offered suggestions to further improve the program for future years, such as including self-reflective exercises on video-recorded teaching performances. As the SAT program is still in its infancy, this feedback is very valuable and is being taken into consideration.

It should be noted that a limitation of the present study includes a possible systematic bias inherent to the self-assessment process. Individuals with little teaching experience may underestimate their self-perceived teaching skills while those with extensive teaching experience may overestimate their abilities. Nevertheless, self-assessment is considered a critical part of establishing competence in medical school as it forces future physicians to reflect on their own strengths and weaknesses and understand which areas to develop in their lifelong medical education [19]. In the future, it may be beneficial to include expert evaluators who can assess the participants’ teaching sessions over time to determine objectively whether teaching competence or performance were improved. It may also be beneficial in future studies to compare these results with students outside of the SAT program as well as recruit a cohort of students with no prior experience or interest in education, and examine whether students gained a greater interest over the course of the program. Similarly, it may be important to compare the extent of previous teaching experience among participants and determine how this may influence their perception of the program.

Finally, as described above, not all participants completed every module or practical teaching session (overall attendance = 82%). Though the participants were not explicitly asked why some sessions were missed, one contributing factor may be associated with students managing other competing demands (e.g. academics or other extracurricular activities). While this is not ideal, it may more accurately represent what a longitudinal program would look like at other institutions where students have educational demands and responsibilities outside of the SAT program. It may also allow students to better understand what it is like to teach while holding other academic and clinical responsibilities.

## Conclusion

We implemented an extracurricular longitudinal 7-month SAT program at the University of Toronto for 20 second-year medical students. This program provided early exposure to medical education theories, and gave students opportunities to apply these theories practically through ongoing teaching and feedback sessions, and reflective exercises. Our findings indicate that students valued the program, saw increases in their teaching and communication skills, and noted improvements in their approach to learning. The instructional format presented here has important implications for undergraduate medical education and may serve as a model for other medical schools that wish to implement similar teaching programs. Offering these opportunities may encourage students to begin developing their teaching skills early on, better preparing them to take on teaching responsibilities as residents and staff physicians.

## Additional files

Additional file 1: SAT program structure. Outline of the dates and topics of each module, practical teaching session, and independent assignments. (DOCX 17 kb)
Additional file 2: Learning objectives for SAT program modules, practical teaching sessions, and independent exercises. (DOCX 19 kb)

## Abbreviations

SAT: Students as teachers
