# Supplementary material for: Development and implementation of a longitudinal students as teachers program: participant satisfaction and implications for medical student teaching and learning
Source: BMC Med Educ. 2017 Jan 31;17:28. doi: 10.1186/s12909-017-0857-8 (PMC5282841; doi:10.1186/s12909-017-0857-8)
Supplement: Additional file 1: — SAT program structure. Outline of the dates and topics of each module, practical teaching session, and independent assignments. (DOCX 17 kb) [file 12909_2017_857_MOESM1_ESM.docx]

**Additional file 1.** SAT program structure

| **MODULE** | **DATE/TIME LOCATION** | **PRACTICAL TEACHING SESSIONS** | **INDEPENDENT ASSIGNMENTS** |
| --- | --- | --- | --- |
| **Module 1**  Meet & Greet;  Teaching Dossier, Orienting the Learner | **Oct. 22, 2014**  5-7pm |  | Observerships (large and small group settings) |
| **Module 2**  Principles of Adult Learning | **Oct. 29, 2014**  5-7pm | Small Group Teaching session #1 Lecture (feedback & video **due Nov. 19, 2014)** |  |
| **Module 3**  Small Group Teaching | **Nov. 19, 2014**  5-7pm | Small Group Teaching session #2 Procedural (feedback & video **due Jan. 19, 2015)** | Reflections due on large and small group observerships (**due Dec. 19, 2014)** |
| **Module 4**  Identifying Learner Needs and Setting Objectives | **January 19, 2015**  5-7pm | Small Group Teaching session #3 (feedback & video **due Feb 18, 2015)** | Clinical observerships (OR and non-OR) |
| **Module 5**  Reflection on experiences so far; Checking In | **February 18, 2015**  5-7pm | Small Group Teaching session #4: Applying teaching skills outside of small group (feedback & video **due Mar. 2, 2015)** | Reflections due on clinical observerships (**due Feb. 18, 2015)** |
| **Module 6**  Effective Feedback | **March 2, 2015**  5-7pm | Large Group Teaching Session: TRI-MEE (feedback & video **due Mar. 2, 2015**) |  |
| **Module 7**  Making Learning Stick | **April 8, 2015**  5-7pm |  |  |
| **Module 8**  Final Debrief; Celebration | **April 20, 2015**  5-7pm |  | Complete teaching dossier (**due Apr. 20, 2015**)  **April 20, 2015**  Final large group debrief session |
