# Supplementary material for: Development and implementation of a longitudinal students as teachers program: participant satisfaction and implications for medical student teaching and learning
Source: BMC Med Educ. 2017 Jan 31;17:28. doi: 10.1186/s12909-017-0857-8 (PMC5282841; doi:10.1186/s12909-017-0857-8)
Supplement: Additional file 2: — Learning objectives for SAT program modules, practical teaching sessions, and independent exercises. (DOCX 19 kb) [file 12909_2017_857_MOESM2_ESM.docx]

# **Additional file 2.** Learning objectives for SAT program modules, practical teaching sessions, and independent exercises

| **MODULE / ACTIVITIES** | **LEARNING OBJECTIVES** |
| --- | --- |
| **Module 1**  Meet & Greet;  Teaching Dossier, Orienting the Learner | • Describe the purpose of a teaching dossier and list the important components of this document  • Implement strategies to accrue contents for their own personal use and to complete their own dossier for submission (tangible outcome)  • Identify components of effective learner orientation  • Implement a strategy for orienting learners to their own learning environment. |
| **Module 2**  Principles of Adult Learning | • Reflect on their identity, roles, and goals as a teacher  • Describe the components of adult learning principles  • Apply adult learning principles to teaching practice |
| **Module 3**  Small Group Teaching | • Identify key elements to effective small group learning  • Discuss the role of the teacher/facilitator in small group teaching  • Apply principles of adult learning to the small group teaching context |
| **Module 4**  Identifying Learner Needs and Setting Objectives | • Describe how societal needs can be established  • Describe how learning needs of students can be determined  • Describe the meaning, inter-relationships, and application of the following concepts: objectives, competencies, milestones, entrustable professional activities  • Write an objective for a teaching session |
| **Module 5**  Reflection on experiences so far; Checking In | • Define “Checking In”  • Discuss the relevance of “Checking In”  • Describe methods of “Checking In” with learners  • Employ behaviours used to “Check In” |
| **Module 6**  Effective Feedback | • List 5 characteristics of effective feedback  • Value the use of a model for giving feedback  • Demonstrate novice level skill when giving feedback to a colleague on a non-medical task |
| **Module 7**  Making Learning Stick | • Discuss challenges associated with teaching in a way that the audience understands and retains the material taught  • Identify and practice teaching strategies that will improve retention  • Reflect on how these strategies can be used in their own educational context |
| **Module 8**  Final Debrief; Celebration | • Describe personal experiences and lessons learned from the SAT program and how this program may have affected their own learning and future career aspirations  • Provide feedback to the SAT program to assist with further development of the program |
| **Practical Teaching Sessions** | • Practice teaching in various contexts (large and small groups) throughout the duration of the program  • Reflect on their progress with each teaching session, how it contributes to their own learning, and how it shapes their own teaching style  • Receive feedback from peers on their teaching |
| **Independent Exercises** | • Appreciate different teaching strategies used in various settings (e.g. classroom, seminars, clinic, OR, etc.)  • Reflect on how these strategies apply to their own teaching approach  • Create a teaching dossier that summarizes their teaching experiences |
